# Microbiological quality of drinking water from water dispensers

**DOI:** 10.3934/microbiol.2025039

**Published:** 2025-12-11

**Authors:** Thomas D. Hile, Raeann Leal, Stephen G. Dunbar, Ryan G. Sinclair

**Affiliations:** 1 School of Science and Health, Crafton Hills College, Yucaipa, CA 92399, USA; 2 Cedars-Sinai Medical Center, Los Angeles, CA 90048, USA; 3 Marine Research Group, Loma Linda University, CA 92350 USA; 4 School of Public Health, Loma Linda University, Loma Linda, CA 92350, USA

**Keywords:** contaminants, disadvantaged communities, outbreaks, microorganisms, water-borne diseases

## Abstract

The consumption of drinking water from sources other than tap water, such as bottled water or water dispenser (WD) machines, is increasing worldwide, driven by consumer preferences for health, convenience, and taste. This trend raises concerns about potential microbial contamination and associated public health risks. In this review, we aimed to comprehensively analyze the scientific literature on microbial contamination in water dispenser machines, evaluate the quality of dispensed water, identify sources of contamination and potential health implications, and propose solutions to mitigate these risks. We conducted a comprehensive search of scientific databases, including PubMed, EBSCO, and Google Scholar, using relevant keywords related to water dispenser contamination. Abstracts and methods of identified studies were critically appraised to ensure rigorous assessment of microbial contamination. Our analysis of approximately 70 studies revealed that despite consumer perceptions of health benefits, water dispenser machines can harbor higher levels of microbial contamination than the tap water sources supplying them. This review underscores the potential public health risks associated with water dispenser use, and highlights the need for increased vigilance, regular maintenance, and further research to ensure the safety of dispensed water.

## 1. Introduction

Access to safe drinking water is a fundamental human right and crucial for public health. While public water systems employ treatment methods, such as coagulation, flocculation, sedimentation, filtration, and disinfection to ensure water safety, there is a growing global trend of consumers turning to alternative drinking water sources, such as bottled water and water dispenser (WD) machines. This shift is driven by consumer perceptions of improved health, convenience, and taste.

The bottled water market has experienced a dramatic surge in recent years, with consumption exceeding 200 liters per capita annually in several countries, including Mexico, Italy, and the United Arab Emirates [1]. In the US alone, bottled water sales reached an estimated $112 billion revenue in 2024 [2].

Water dispensers, also known as water coolers, dispense drinking water and are broadly classified into point-of-use (POU) and bottled WDs. Point-of-use WDs are directly connected to the tap water supply, while bottled WDs use replaceable water bottles. Many WDs incorporate additional filtration technologies, such as reverse osmosis, carbon filtration, and UV sterilization, to further purify the water and remove unwanted tastes or odors [3]. Despite these filtration efforts, concerns remain about the potential for microbial contamination in WDs. Sporadic reports of contamination in both bottled water and WDs have raised public health concerns. Microbial contamination in drinking water can lead to waterborne diseases and outbreaks, posing significant health risks to consumers [4].

In this review, we aimed to systematically analyze the scientific literature on microbial contamination in WDs, evaluate the quality of dispensed water, identify sources of contamination and potential health implications, and propose solutions to mitigate these risks. To achieve this, we conducted a comprehensive search of relevant scientific databases using keywords related to WD contamination. Our analysis was guided by key research questions, including: What are the regulations governing WDs? How effective are the filtration technologies used in WDs? Is water from WDs more contaminated than tap water? What are the common contaminants found in WDs? And what measures can be taken to prevent and control microbial contamination in WDs?

## 2. Materials and methods

We aimed to analyze the existing literature on microbial contamination of WDs. A systematic search strategy was employed to identify relevant published studies. The search strategy involved a three-step approach. Initially, a preliminary search was conducted using Google and Google Scholar to gain an overview of the available literature. Afterward, a comprehensive search was performed in PubMed, EBSCO, and Google Scholar using relevant keywords and index terms related to water dispenser contamination. Keywords were adapted as necessary for each database. The reference list of all included sources of evidence was screened for additional studies. We excluded studies published in languages other than English.

All identified citations were collected and managed using Endnote X9.3.3 to facilitate the removal of duplicate entries. Following a pilot assessment, titles and abstracts were screened to determine their relevance based on pre-defined inclusion criteria. The full text of potentially relevant studies was retrieved for further evaluation if they answered one or more of our research questions. A data extraction process was conducted to gather relevant information from the included studies. This included international laws relevant to contamination, health indicators, the physical condition, the detection of microbes, and the concentrations of microbes. These are in the tables below.

Following the search, all identified citations were collected and uploaded into Endnote X9.3.3, and all duplicates were removed [5]. Following a pilot test, titles and abstracts were screened by reviewers (i.e., us). We provide a summary of the studies that (1) reported on the drinking water quality of WDs, (2) assessed how well the subject represented the scientific literature, (3) described the health risks associated with WDs, and, (4) included a quality control.

## 3. Results and discussion

To address the research question regarding the types of contaminants present in WDs, we identified fecal coliform and *P. aeruginosa* as recurring contaminants in these systems. In response to whether regular maintenance helps reduce biofilms in WDs, the evidence indicates that routine cleaning with disinfectants significantly decreases bacterial contamination. While WD manufacturers recommend regular maintenance and timely filter replacement, delays in replacing filters can turn them into breeding grounds for microbes. This underscores the importance of proper maintenance in minimizing microbial contamination in WDs.

### 3.1. Regulation of WDs worldwide

International water quality monitoring programs have been implemented to ensure public health safety from waterborne diseases. However, since WDs are typically connected to reliable, well-regulated, and monitored tap water, lawmakers assume potable and safe water quality from WDs, and that dispensed water is typically free from contaminants [6]. Moreover, WDs provide additional filtration and water treatment layers that presumably should result in potable, risk-free drinking water. In Italy, drinking water, including water from water vending machines WVMs, must be free from any pathogenic microorganisms and chemical contaminants that may endanger human health [7]. The lack of WD regulations has been noted in parts of the world. In Malaysia, for example, the National Water Quality Standards, Ministry of Health, regulates the quality of drinking water and stipulates that a ‘Class I’ water quality should contain total coliform at a maximum level of 100 CFU/mL with fecal coliform at a maximum of 10 CFU/mL [8] . In Switzerland, drinking water is regulated for treated and untreated water systems, such as containers, bottles, and mineral water for ice water added to beverages [9]. Nevertheless, for soda fountains, there are no manufacturer guidelines for maintenance in Switzerland other than a monthly cleaning recommendation for nozzles and pipes [9]. Additionally, no regulations for microbiological limits were set for WDs. Similarly, Zanetti et al. [10] and Liguori et al. [7] in Italy indicated that there were no regulations for microbial contamination of drinking water from WDs. The European Community Directive Guidelines require drinking water for human consumption, including WDs, to be free from any pathogenic and chemical contaminants [11]. However, there is no mandatory European Union quality standard for drinking water for WDs [10]. Table 1 summarizes the laws and ordinances of WDs in some countries worldwide. In Table 2, we present US-based laws and ordinances that provide oversight of drinking water from WDs.

**Table 1.** International laws and ordinances for drinking water quality from WDs.

Location	Ordonnance	Type of requirement	Enforcement
UK	Water Supply (Water Quality) Regulations 2016 Health and Safety at Work Act 1974 Food Safety Act 1990 and Food Hygiene Regulations 2006 Drinking Water Inspectorate (DWI) Guidelines British Water Cooler Association (BWCA) Standards Local Health Authorities	Bottled water is considered a food product and should be treated as such during the bottling, storage, delivery, and dispensing processes. Regular sanitization is required for all water coolers. Bottled water dispensers should be sanitized every 3 months, while plumbed-in water coolers require sanitization every 6 months.	Failure to comply with these regulations can lead to enforcement actions by regulatory bodies, including fines, orders to remedy deficiencies, or other legal actions to protect public health. Local authorities and the DWI play key roles in monitoring compliance and enforcing these standards.
Canada	Health Canada, Provincial and Territorial Governments Minister of National Health and Welfare ARI Standard 1010-82	Water dispensers must undergo regular testing for contaminants to ensure the water meets safety standards. Dispensers need to be regularly cleaned and maintained to prevent contamination and ensure hygienic conditions.	Local health authorities and provincial/territorial regulatory bodies conduct inspections to ensure compliance with regulations. Non-compliance can result in enforcement actions, including fines, orders to remedy deficiencies, and other legal penalties to protect public health.
EU	Regulation (EC) No 178/2002 of the European Parliament and of the Council Drinking Water Directive (DWD) 98/83/EC Directive (EU) 2020/2184 Regulation (EC) No 1935/2004 on Materials and Articles Intended to Come into Contact with Food	For water intended for human consumption that is bottled or contained for sale, or used in the manufacture, preparation, or treatment of food, it must adhere to this Directive until the point of compliance, namely the tap. After this point, if the water is intended or reasonably expected to be ingested by humans, it should be considered as food.	Each member state has designated authorities responsible for monitoring compliance with water quality standards. These authorities conduct inspections and audits of water dispensers in public places, workplaces, and businesses. Non-compliance with regulations can result in enforcement actions, including fines, orders to correct deficiencies, and other legal penalties to protect public health.
Malaysia	Ministry of Health and the water purveyor (Food Act 1983 and Food Regulations 1985) National Water Services Commission (SPAN)(Water Services Industry Act 2006 (Act 655)) Department of Environment (DOE) (Environmental Quality Act 1974) Malaysian Standards (MS) Certification and Best Practices	The drinking water quality standards are applicable to all water intended for human consumption. This includes drinking water from all public water supply systems, tank supplies and water used for bottled drinks and ice manufacturing. Drinking water must be clear and free from any unpleasant taste, color, or odor. It should be enjoyable to drink and free from harmful organisms, chemical substances, and radionuclides in amounts that could pose a health risk to consumers. The quality of drinking water is measured in terms of its microbiological, physical, chemical and radioactivity characteristics.	The Ministry of Health and SPAN conduct regular inspections and audits of water dispensers in public places, workplaces, and businesses to ensure compliance with regulations.
Switzerland	Swiss Food Law Ordinance on Drinking Water Federal Office of Public Health (FOPH) Swiss Drinking Water Directive	all foodstuffs, including drinking water, are safe for consumption. aligns with European Union standards but may include stricter national requirements	In addition to federal regulations, cantonal and municipal authorities may have additional rules and standards that must be followed. These can vary by region and may include specific requirements for the installation, maintenance, and monitoring of drinking water dispensers.

**Table 2.** The US-based laws and ordinances that provide oversight of drinking water to WDs.

Rule	Government	Description	Regulations for WDs	Reference
SDWA	USEPA	Originally passed by Congress in 1974 to protect public health, the law regulates the nation's public drinking water supply. It was amended in 1986 and 1996 to include measures to safeguard drinking water and its sources, such as rivers, lakes, reservoirs, springs, and groundwater wells. However, the SDWA does not regulate private wells serving fewer than 25 individuals.	None	[12]
NPDWS	USEPA	The National Primary Drinking Water Regulations (NPDWR) are legally enforceable standards and treatment techniques applicable to public water systems. These primary standards and treatment techniques protect public health by limiting contaminant levels in drinking water.	None	[13]
NSDWS	USEPA	NSDWRs (or secondary standards) are non-enforceable guidelines regulating contaminants that may cause cosmetic effects (such as skin or tooth discoloration) or aesthetic effects (such as taste, odor, or color) in drinking water. EPA recommends secondary standards to water systems but does not require systems to comply with the standard. However, states may choose to adopt them as enforceable standards.	None	[14]
CGMP	USFDA	CGMP stands for Current Good Manufacturing Practice regulations enforced by the FDA. These regulations ensure that manufacturing processes and facilities are properly designed, monitored, and controlled. By adhering to CGMP, drug manufacturers can guarantee the identity, strength, quality, and purity of their products. This involves implementing strong quality management systems, sourcing high-quality raw materials, establishing effective operating procedures, detecting and investigating quality deviations, and maintaining reliable testing laboratories. Drinking fountains and coolers must be made from impervious, non-oxidizing materials and designed for easy cleaning. The jet of a drinking fountain should be angled, with a protected orifice to prevent contamination from mouth droplets. The jet orifice must be positioned high enough above the basin rim to prevent backflow. (b) Ice should not come into contact with water in coolers or constant temperature bottles. (c) Constant temperature bottles and other containers used for storing or dispensing potable water must always be kept clean and subjected to effective bactericidal treatment after each occupancy of the served space and at least once a week.	Yes	[15]

### 3.2. The use of water dispensers over tap water

Reasons why people prefer to drink water from sources other than tap water are mainly subjective and range from taste, convenience, and accessibility to financial stability, lifestyle, and health-related concerns. Another reason for the success of WDs is the consumer targeting strategies used by vendors. It has been demonstrated that WVMs are generally in low-income and immigrant communities [16],[17]. These communities are generally comprised of immigrants who believe tap water is unsafe for consumption and, therefore, the only suitable drinkable water is from sources other than in-house taps. Due to their low-income status, many people in such disadvantaged communities prefer to purchase bulk water from WVMs, which are less expensive to access than bottled water. Cristobal et al. [3] noted that people preferred WDs over tap water for real and perceived health issues. The booming drinking water market offers options with various health claims, such as reduced aging, prevention of some chronic diseases, such as cancer, and improved body pH and function [18]. The few researchers addressing the microbial contamination of drinking water from WDs (Table 3) have concluded that WDs were more contaminated than the tap water that supplied them. Despite limited research to support such claims, many people worldwide, desiring to improve their health, make substantial investments in the quality and brand of drinking water. Table 3 presents studies that compared WDs to tap water. With usage and lack of maintenance, WDs become more contaminated than tap water from municipal water districts to which they are connected. Raymond et al. [18] showed that carbon filters in point-of-use (POU) devices resulted in 1-2-fold increases in bacterial concentrations over those found in public tap water samples. After WD treatment and flushing, it has been shown that WDs still harbor microorganism that regrow following WD treatments. In Switzerland, Baumgartner et al. [6] found that WDs mainly caused water contamination and that bacterial populations increased after a week of proper maintenance and treatment. Since most studies on microbial contamination of WDs recommend regular maintenance and cleaning [19]–[21], the recurrence of microbes after a few days of maintenance is counterintuitive and weakens the assumption that well-maintained WDs are free from microbes. For ice dispenser machines in Turkey, Hampikyan et al. [22] found ice contamination with coliforms, and *E. coli* was linked to the ice machines themselves. A study by Precha et al. [23] in Thailand found that water dispensers in higher educational institutions had higher bacterial contamination than the water that supplied them. Additionally, Farhadkhani et al. [24] found that the bacteriological water quality of WDs in Iran was more contaminated than the water supplying the WDs. Furthermore, Lévresque et al. [25] showed that WD microbial contamination in Canada was due to the water coolers rather than the water sources. They concluded that municipal tap water was superior in quality to water coolers. In Italy, Zanetti [10] et al. found heterotrophic plate counts (HPCs) in water collected from dispensers were higher than those of tap water supplying WDs. They concluded that bacterial regrowth seen in WDs was due to bacterial build-up (biofilms) in WDs. Biofilms provide a reservoir of constant contamination for water dispensers due to the biofilm's ability to leach planktonic cells and by-products into the water [10]. In Malaysia, the significant dependability of drinking water from WDs was considered high [26], although water quality may be compromised due to contaminants found therein. Chaidez et al. [3] indicated that water quality deteriorated rapidly due to the daily use of WDs.

Similarly, Baumgartner et al. [6] in Switzerland found that aerobic plate counts increased in coolers compared to the supplying source. Likewise, Lévresque et al. [25] found that WDs were more contaminated than the water supply sources in Québec, Canada. In Italy, Liguori et al. [25] in their study on the microbiological quality of drinking water from dispensers, found that none of the tap water samples had microbial contamination greater than WDs, and no coliforms were found in the tap water. Therefore, evidence suggests that tap water is not generally the immediate cause of WD contamination; rather, the likely cause is the formation of biofilms in machines. Additionally, Zanetti et al. [27] stipulated that WDs favor the multiplication of heterotrophic bacteria and that *P. aeruginosa* was recurrent in WD systems.

Based on these studies, we conclude that using WDs is not necessarily safer than tap water since studies have demonstrated that WDs are more contaminated than tap water. These studies have proposed regular cleaning and flushing of WDs as the best solution to reduce microbial colonization and bacterial regrowth in WDs.

**Table 3.** Comparative studies of contamination of tap water versus WDs.

Study Location	Organism	WDs	Tap	References
Brazil	Coliforms	76.6%	36.4%	[28]
Quebec, Canada	Coliforms	28%	22%	[25]
Iran	HPC	62%	61%	[24]
Switzerland	*P. aeruginosa*	24.1%	10%	[24]
Germany	*P. aeruginosa*	50%	9%	[29]
Italy	*P. aeruginosa*	28.9%	1%	[7]

### 3.3. Biofilms

Biofilms are the predominant mode of microbial growth in drinking water distribution systems. These biofilms utilize extracellular polymeric substances that protect the microbial biomass from environmental stressors. The formation of biofilms in WDs is supported by many factors, including WD lines and connections, water stagnation, surface-to-volume ratio, and the absence of chemicals, such as free chlorine capable of controlling bacterial regrowth. Biofilm formation is a considerable problem for the water industry due to the ability of biofilms to shelter microorganisms and play a role in bacterial contamination, affecting the aesthetic parameters of potable water [30]. Several researchers have found that WDs are highly affected by biofilm formation. In Poland, for example, Szymanska et al. [31] found that biofilms constitute a significant problem in environmental, industrial, and medical settings. These biofilms can harbor undesirable levels of opportunistic or frank pathogens and facilitate their infiltration into drinking water [27],[30],[32]. As described by Liguori et al. [7], the contamination of WDs and biofilm formation is a gradual process, beginning with the accumulation of small numbers of microorganisms.

Although WD manufacturers require regular maintenance and filter replacement, filters may not be replaced on time and, therefore, become a breeding source for microbes. Activated carbon filters used to filter water in some WDs may amplify the bacterial population in tap water, especially during stagnation periods, if the filtration capacity [33] is reached. Surfaces of WDs enable biofilm attachment and proliferation. Waterlines made of plastic allow microorganisms to adhere to the internal surfaces of the tubes, favoring biofilm formation [34],[35]. Similarly, Szymanska et al. [36] found that materials, such as ethylene-propylene and latex surfaces, are more suitable for bacterial growth than glass or stainless steel surfaces. Buffet-Bataillon et al. [37] reported that rubber-lined hoses containing elevated levels of plasticizer favor bacterial growth and that bacteria attach predominantly to rough surfaces. Consequently, WDs are highly vulnerable to adequate biofilm formation.

Another suitable breeding surface for biofilms are dispenser spigots, which are typically a narrow-bore water line made of plastic material. In 1987, Robertson et al. [38] sampled nozzles from hot-drinks vending machines in Italy and found they were 100-fold more contaminated than other areas of water dispensers. Another pilot study by Cardici et al. [39] in Italy also found that nozzles were more contaminated than other surfaces of the machines. Consequently, this can lead to the transfer of microbial contaminants to humans, causing gastrointestinal illnesses [39]. Hunter et al. [40] indicated that inadequate WD cleaning may result in biofilm formation, contributing to bacterial community growth in water samples. Biofilm formation may also be exacerbated by exposure of drains to fomites [41]. In most studies, dispenser nozzles were swabbed along the inside surface and later used for bacteriological analysis. Muhammad et al. [40] identified microbial contamination in WDs in Malaysia, with bacterial colonies found in all nozzles. They concluded that cleaning and maintenance of the WDs is imperative to maintaining the quality of the water supplied. Cardaci et al. [39], showed that swabbed inner nozzles of hot beverage vending machines in Italy were significantly more contaminated than outer surfaces of dispensers.

### 3.4. Health-related issues associated with WDs contamination

The EPA has selected a list of microorganisms, termed (microbial indicators) that help determine drinking water quality. Fecal Indicator Bacteria (FIB) and coliforms are indicators used to assess the microbiological contamination of water and evaluate compliance with water regulations. Total coliforms are generally used as indicator organisms of the presence of human and animal feces, and may be present in insect feces [3]. They are a group of related bacteria found in soil, aquatic environments, vegetation, and human or animal waste that are mainly unharmful to humans [35]. The EPA also considers total coliform indicators of other pathogens in drinking water, such as bacteria, parasites, and viruses [42]. Additionally, municipal water districts also use coliforms to measure water treatment efficiency and the integrity of distribution systems [42]. Identifying coliform in drinking water suggests the potential presence of pathogenic microorganisms, such as *Salmonella spp*., *Shigella spp*., and *Vibrio cholerea* [28]. Säve-Söderbergh et al. [43], in their study on gastrointestinal illnesses linked to incidences of drinking water contamination in Sweden, found elevated risks of vomiting and acute gastrointestinal illnesses related to water contamination. Moreover, Jakopanec et al. [42], Laine et al. [45], and Widerstrom et al. [44] presented evidence of considerable waterborne outbreaks in the Nordic countries. In Brazil, coliform bacteria are the only ones regulated by law in tap and bottled water [31]. Although the presence of coliforms in drinking water may not be an immediate threat or public health concern, its presence should always be taken seriously as an indicator of fecal contamination [28]. In the US, the Revised Total Coliform Rule (RTCR), which is committed to increasing public health protection by reducing potential pathways of entry for fecal contaminants into water distribution systems, set a maximum contaminant level goal (MCLG) of total coliforms, including fecal coliform and *E. coli*, to zero, with a maximum contaminant level of 5% [42]. A study in the US by Cristobal et al. [3] revealed that 20% of samples had total coliforms. Moreover, in Malaysia, Ang et al. [3] found that 80% of WVMs were contaminated by coliforms resulting from filtration failures. However, the inconsistent use of FIBs and total coliforms can prevent accurate data analysis and evaluation of the severity and disease burden in affected locations [53]. We present Table 4 to summarize WDs contamination, microorganism indicators, health implications, and recommendations of selected studies.

We decided to include *P. aeruginosa* as an indicator of water quality because many laboratories systematically screen for *P. aeruginosa* to assess water quality. *P. aeruginosa* is of particular interest in this review due to the seriousness of its infection, with the potential to cause mortality and morbidity in immunocompromised people [46]. The presence of *P. aeruginosa* in WDs has been reported by researchers worldwide and has always received special attention due to its ubiquitous nature and its role in nosocomial infections. As part of a large group of free-living bacteria that are abundant in the environment and often found in natural waters, such as lakes and rivers, *P. aeruginosa* is rarely found in drinking water, where its occurrence is related to its ability to colonize biofilms in plumbing fixtures, such as faucets and showerheads [32]. This microorganism is known to cause endocarditis, folliculitis, keratitis, cystic fibrosis (CF), osteomyelitis, and is a leading cause of septicemia and illness in immunocompromised individuals [47]. It is also a known opportunistic pathogen that may be found in drinking water. With its low nutritional needs, the bacteria can multiply quickly, reaching a level that may represent a health risk to humans, especially children, the elderly, and the immunosuppressed [29],[48]. A similar study in Malaysia by Chaidez et al. [3] found that 23% of WVMs had *P. aeruginosa* and demonstrated that colonization of WDs by this species is intransient due to its ability to form biofilms in pipes [49]. Although a different species of the genus *Pseudomonas*, Wong [50] et al. found a source of the spread of *Pseudomonas fluorescens* in hospital bone marrow transplant units in the UK was contaminated drinking water from dispensers [46].

Opportunistic pathogens can become infective after their host's immunity is compromised [51]. Many pathogenic microorganisms are considered opportunists because, although capable of causing human disease, they are zoonotic and opportunistic in numerous other hosts. These opportunists can emerge from among the ranks of normally commensal symbionts, such as *Streptococcus pneumonia* and *Staphylococcus aureus* [52]. In a study on soda fountain machines by White et al. [53] in the US, they found that 17% of bacteria, such as *Chryseobacterium meningosepticum*, *Klebsiella, Staphylococcus, Stenotrophomonas, Candida*, and *Serratia*, were identified as opportunists. Those authors concluded that soda fountain (SF) machines may harbor opportunistic pathogens, which may then cause episodic gastrointestinal infections among consumers and potentially cause serious health risks in immunocompromised individuals. Another group of bacteria found in all types of water is heterotrophic plate count (HPC) bacteria.

**Figure 1.**
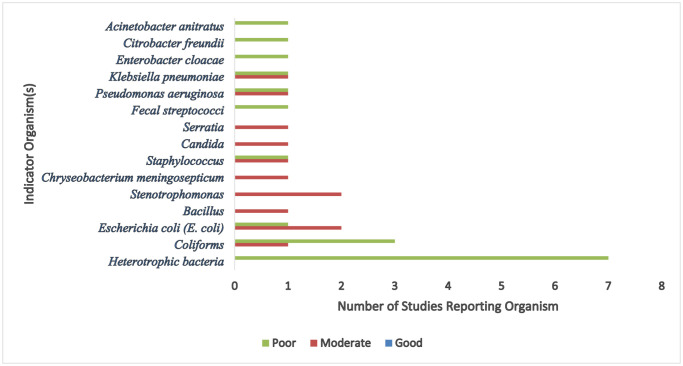
Distribution of microbial indicator organisms by water quality classification.

Heterotrophic plate count bacteria are classified as oligotrophic or opportunist [54] microorganisms and are commonly used to measure the general microbiological quality of drinking water. The EPA recommended that HPC bacteria not exceed 500 CFU/mL in drinking water. This recommendation also aims to limit interference with the detection of coliform bacteria [55]. The World Health Organization (WHO) states that coliform testing methods are better indicators than HPCs for determining water quality [56]. In contrast, a study by Smith et al.[10] on drinking water evaluation in Canada indicated that HPC results were not water quality or safety indicators. Nevertheless, the EPA and other water governing bodies worldwide continue to use HPCs as water quality indicators. Studies have revealed that HPC bacteria may not be as harmless as once considered, with only disease-weakened individuals at risk of contamination [57]. Few studies have reported the presence of HPCs in WDs. Farhadkhani et al. [24] found that 62% of the water sampled from WDs in Iran had HPC above 500 CFU/mL. Also, Cristobal [3] et al. found HPC bacteria in all 30 samples studied in the US, and 73% had HPCs counts above 500 CFU/mL, with HPC bacteria on the dispensing nozzle ranging from 9 to 48000 CFU•EA^−1^ [3]. Another study in the USA by Hile [58] et al. revealed 32% of WVMs had total coliforms, and 21% had HPC above the 500 CFU set by the Environmental Protection Agency. Hunter et al. [39] found that 84% of WVMs and 39% of soft drinks sampled had HPCs above the UK's EPA recommendation. In Brazil, Da Silva et al. [28] found that 87% of samples had HPC in WDs above the HPC threshold for Brazil, which is also 500 CFU/mL. Worldwide, HPCs above the recommended threshold reinforce the idea that WDs are potential breeding sites for bacteria, including pathogenic microorganisms that may severely impact public health. Figure 1 illustrates the frequency of microbial indicator organisms (e.g., *E. coli*, coliforms, *Pseudomonas aeruginosa*, *Staphylococcus*, *Bacillus*) reported across studies, categorized by overall water quality levels (good, moderate, or poor). The data reflect the number of studies in the review that identified each organism within the specified water quality category. Studies listed in the category were those that stated they were above the EPA limits and those listed as moderate were stated as present in the tested samples.

**Figure 2.**
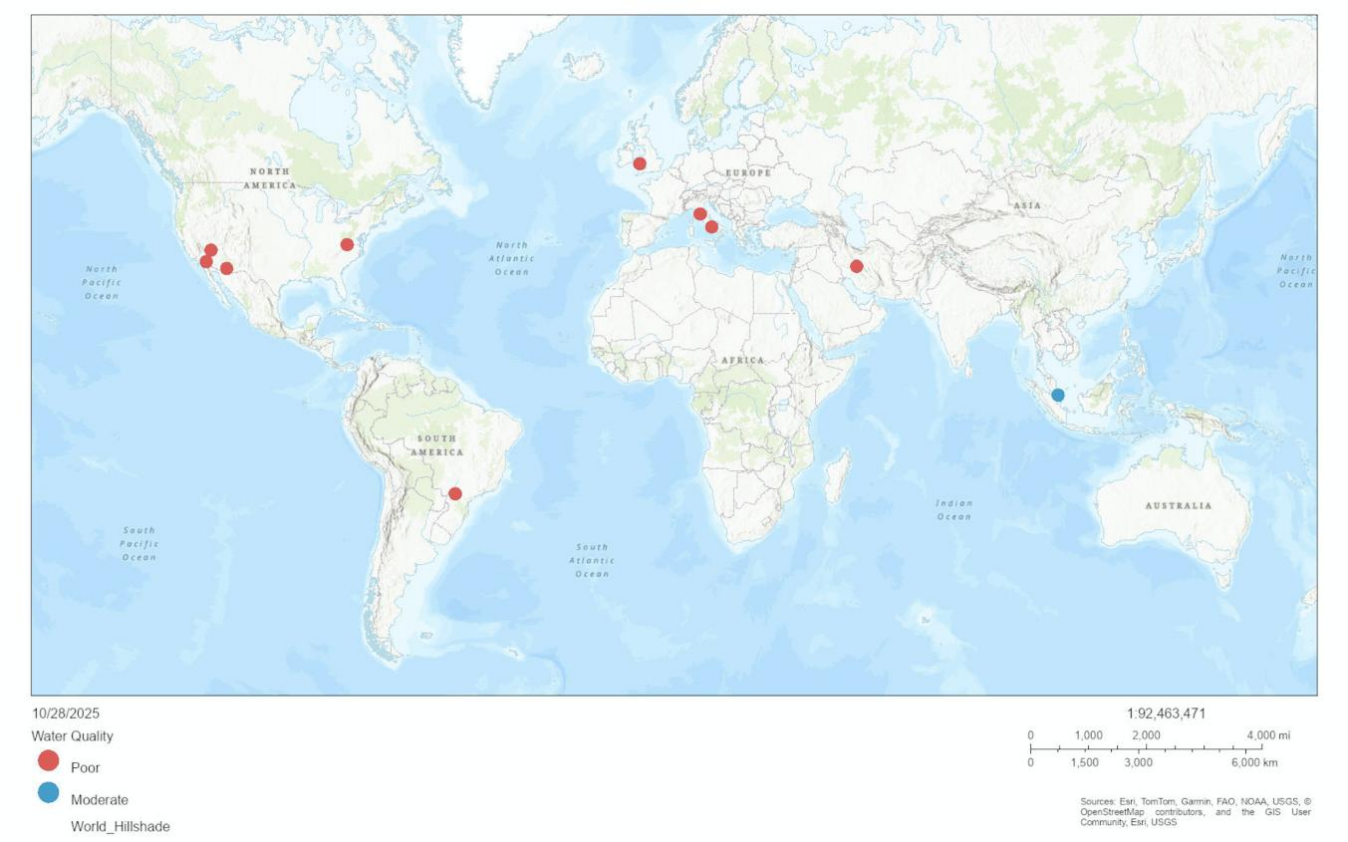
Geographic distribution and corresponding water quality classification of vending machines, water dispensers, and beverage machines across global study sites.

Figure 2 presents the geographic distribution of studies evaluating microbial contamination in water vending machines (WVMs), water dispensers (WDs), and beverage machines. Each point on the map represents a study location, color-coded by overall water quality classification (good, moderate, or poor). The visualization was created using ArcGIS Online, with coordinates derived from published study locations to illustrate global patterns in water quality and contamination levels.

**Table 4.** Global overview of water quality and microbial contamination in water vending machines (WVMs), water dispensers (WDs), and beverage machines

Source	Location	Quality of Drinking Water	Indicator organism(s)	Health Implication	Recommendations	References
Hot drink WVMs	Siena, Tuscany, Italy	Bacterial counts exceeded permissible limits across all culture media.	Heterotrophic bacteria (indicator organisms not specified)	Given the high number of CFU.cm^2^, WVMs may pose a potential health risk for consumers.	Further studies are recommended to assess contamination sources and preventive measures.	[59]
WVMs	Tucson, Arizona, USA	Heterotrophic plate count (HPC) bacteria were detected in all samples, with 73% exceeding 500 CFU·mL. Total coliforms and *Escherichia coli* were also identified.	HPCs, *Coliforms, E. coli*	Poor hygienic conditions of water dispensers pose potential health risks to consumers.	Routine monitoring, proper maintenance, and increased public awareness about dispenser hygiene are recommended.	[3]
WVMs	Eastern Coachella Valley, California, USA	32% of WVMs contained total coliforms, and 21% exceeded EPA heterotrophic plate count (HPC) limits. Genetic material from pathogenic microorganism was also detected.	HPCs, *Coliforms*	Detection of pathogen-associated genetic material indicates potential public health risk.	Regular flushing of drains and pipes and monthly cleaning of nozzles are recommended to reduce biofilm formation and microbial concentrations. Use of nanoparticle-coated hoses my further help control microbial growth.	[58]
WVMs	Johor Bahru, Johor, Malaysia	Presence of bacterial genera including *Pseudomonas*, *Bacillus*, and *Stenotrophomonas* indicates microbial contamination.	*Pseudomonas*, *Bacillus, Stenotrophomona*.	Detection of opportunistic bacteria suggests possible risks for immunocompromised consumers.	Regular servicing and maintenance of WVMs are recommended to minimize microbial contamination and ensure water safety.	[26]
Ice, soda, and WDs	Las Vagas, Nevada, USA	3.3% of ice samples and 55.6% of soda samples exceeded the EPA limits for heterotrophic plate count (HPC) bacteria in drinking water.	HPCs	Elevated HPC levels indicate potential microbial contamination that could have public health implications.	Routine monitoring and maintenance of beverage dispensers are recommended to ensure compliance with bacteriological quality standards.	[51]
Soda fountain machines	Roanoke Valley, Virginia, USA	*Coliform* bacteria were detected in 48% of the beverages, and 20% exceeded 500 CFU·mL for heterotrophic plate counts. *E. coli* was found in 11% of samples, and over 17% opportunistic pathogens such as *Chryseobacterium meningosepticum, Klebsiella*, *Staphylococcus*, *Stenotrophomonas*, *Candida*, and *Serratia* were also isolated.	HPCs, *Coliforms*, *E. coli, Chryseobacterium meningosepticum, Klebsiella*, *Staphylococcus*, *Stenotrophomonas*, *Candida*, and *Serratia*.	Presence of potentially pathogenic microorganisms may cause gastric distress in the general population and could pose greater risks to immunocompromised individuals.	Enforcement of regulations and strict hygienic practices for soda fountain maintenance are recommended to reduce microbial contamination.	[60]
Water coolers (carbonated and non-carbonated)	Naples, Italy	Bacterial counts at 22 °C and 37 °C exceeded required limits in 71% and 81% of non-carbonated samples and in 86% and 88% of carbonated samples, respectively.	HPCs	Elevated heterotrophic bacteria levels indicate potential public health risks from inadequate dispenser hygiene.	Routine monitoring and maintenance of water coolers are recommended to ensure compliance with microbiological safety standards.	[7]
WDs and municipal tap water	Maringa, Parana, Brazil	36.4% of municipal tap water samples and 76.6% of the 20 L bottled water samples from water dispensers were contaminated.	Total coliforms, thermotolerant coliforms, *E. coli*, fecal *streptococci*, *Pseudomonas aeruginosa*, *Staphylococcus spp*., and heterotrophic plate count (HPC).	Detection of multiple bacterial indicators, including potential pathogens, indicates a significant public health concern.	Strengthening surveillance of bottled water industries and conducting routine *Pseudomonas* testing within municipal water systems are recommended to ensure safe drinking water.	[28]
WDs	Wales, UK	84% of the water samples and 39% of drink samples were unsatisfactory or exceeded the UK EPA limits for heterotrophic plate counts (HPC).	*Klebsiella pneumonia, Enterobacter cloacae, Citrobacter freundii, and Acinetobacter anitratrus*.	Although no disease outbreaks were reported, the presence of opportunistic bacteria indicates potential health risk from poor dispenser hygiene.	Routine maintenance and strict hygienic control of water vending machines are recommended to limit bacterial growth and ensure adequate water quality.	[39]
WDs	Isfahan, Iran	62% of samples had HPCs above recommended limits.	Heterotrophic bacteria	Detection of opportunistic bacteria in water coolers indicates potential public health risks.	Implement periodic disinfection procedures and establish routine monitoring systems for water coolers to ensure safe drinking water.	[24]

**Table 5.** Regular treatment and maintenance decrease the amount of biofilms.

Study Location	disinfectants	Organism	Findings	References
Italy	Peracetic acid (PA) and hydrogen peroxide (HP)	HPC	HP was more effective in controlling bacterial contamination and regrowth in WDs	[8],[10]
Switzerland	pasteurization	*P. aeruginosa*	To control the regrowth of *P. aeruginosa* after two weeks	[6]
Korea	Plasticizer & antimicrobial coating content	HPC	Bacterial growth inhibition	[61]
USA	HP; colloidal silver; buffered pH	*Ralstonia pickettii, Burkholderia multivorans, Caulobacter vibrioides*, and *Cupriavidus pauculus*	HP and 400 ppb and colloidal silver effectively reduced the bacterial concentrations to less than 1 CFU for up to 2 months.	[50]

### 3.5. Regulation of WDs in the US

The US Congress passed the Safe Drinking Water Act (SDWA) in 1974 and amended it in 1986 and 1996 to ensure that drinking water piped into American households was safe and free from contaminants [62]. The US EPA is charged with setting standards for drinking water quality and monitoring States and local authorities to enforce those standards. To ensure the safety of drinking water, the EPA has set maximum contaminant levels and treatment requirements for more than 90 contaminants in public drinking water. Moreover, the National Primary Drinking Water Regulations (which must follow regulations by the water system) and the National Secondary Drinking Water Regulations (for water issues unrelated to health, such as taste and color) have set additional guidelines that States may adopt and enforce [61]. However, the high water-quality standards set by the EPA do not include bottled water or WDs. The Food and Drug Administration (FDA) regulates and monitors the quality of drinking water for bottled water and WVMs [61], and sets the Current Good Manufacturing Practices (CGMP) that require bottled water to be transported under sanitary conditions and protected from bacteria, chemicals, and other contaminants using quality control processes and sample testing [61].

Additionally, the FDA monitors bottled water products and processing plants by inspecting sanitizing and bottling procedures and ensuring that companies analyze source and product water for contaminants. Under the 1978 Vending of Food and Beverage Model Ordinance, the FDA regulated machine construction materials, yet did not (and still does not) ensure the quality of the water product [63]. The Code of Federal Regulations: Title 21 [61] stipulates that WDs constructed of waterproof and nonoxidizing material, which cannot be easily cleaned, have the jet of the WD slanting with the orifice above the rim to prevent backflow [64]. Additionally, containers and storage bottles should be kept clean and subject to efficient bactericidal treatment weekly [64]. In California, the Health and Safety Code (H&SC), Section 111120, [76] requires that WD operators obtain an operator license issued by the California Department of Public Health (CDPH), Food and Drug Branch (FDB) that should be renewed annually [65]. However, routine maintenance and treatments have no enforced regulations and inspections. There are no regulations for WDs, such as daily testing, monitoring physico-chemical parameters, coliforms, machine cleaning, or other regulatory measures that are typically applied to municipal water supplies. Nevertheless, there are requirements and directions to maintain the machines.

### 3.6. Microbial contamination of Soda Fountains

A study in the US by White et al. [66] revealed that 48% of beverage samples from SFs had coliforms, and 20% had HPC above 500 CFU·mL^−1^. The same study showed that more than 11% of the beverages analyzed had *E. coli*, and 17% had *Chryseobacterium meningosepticum* and other opportunistic pathogenic microorganism species, such as *Klebsiella, Staphylococcus, Stenotrophomonas, Candida*, and *Serratia*. In 2006, a study in Germany by Chaberny et al. [33] showed that water from a hospital soda dispenser did not meet the German requirement for water quality after being tested weekly and was required to be removed from the high-risk wards of the hospital. Another study in Las Vegas by Hertin et al. [64] found that 55% of soda samples exceeded the limits set by the EPA for HPC, with 88% of the same soda dispenser samples having coliforms. The presence of microbial contaminants in soda fountains should not be overlooked. The lack of a residual disinfectant such as chlorine in WDs allows microorganisms to regrow downstream of filtration. Unlike municipal water distribution, where residual chlorine suppresses microbial proliferation, WDs supply water that is typically free of disinfectant. This creates favorable conditions for microbial survival, particularly within internal tubing and dispensing outlets. Furthermore, periods of water stagnation within the storage reservoir or internal lines enhance the adhesion of microorganisms to surfaces, providing a foundation for biofilm development and microbial amplification [33],[54],[67].

### 3.7. Solutions to WD contamination

Most researchers who evaluated microbial contamination from WDs concluded that the solution to substantially reducing bacterial contamination was through regular cleaning and maintenance of water dispensing machines. Zanetti et al. [10] used disinfectants to clean WDs and found effective reductions in bacterial concentration. Baumgartner [6] et al. suggested that WDs be adequately maintained and proposed pasteurization and microfiltration to solve the problem. However, maintenance of WDs has been demonstrated to be a short-term solution. For example, a study by Zanetti [10] in Italy revealed that WDs treated with peracetic acid (CH_3_CO_3_H) and hydrogen peroxide (H_2_O_2_) disinfectants effectively eliminated psychrophilic HPC. A few days after disinfection, however, they observed bacterial regrowth. Sacchetti [68] et al. found that bacterial populations regrew after treatments in a similar study on WDs. They also found that treatment time was essential in reducing the regrowth of bacteria in the WDs. In Malaysia, Ang [69] et al. found no significant correlation between maintenance status and the presence of total coliform. Consequently, cleaning and treating WDs is not a long-term sustainable solution to the problem of water contamination in WDs. Table 5. presents the effect of regular treatment and the reduction of biofilm formation. Regular disinfection frequency plays a critical role in preventing microbial regrowth and biofilm formation inside WDs. Evidence suggests that internal system lines and dispensing outlets should be disinfected every two to four weeks, depending on unit usage and environmental conditions [10],[54]. In high-use locations, more frequent sanitation schedules may be required every week due to increased risk of biofilm formation, as lack of routine maintenance has been consistently associated with elevated contamination in WDs [32],[35],[50].

### 3.8. Scarcity of studies on WDs

In prior literature, issues related to WD contamination mostly reported the regrowth of microorganisms and biofilm formation. Microbial contamination of WDs and biofilm formation represents a serious microbial risk that requires further assessment using advanced techniques in molecular biology. In 1999, Cristobal [3] et al. noted the scarcity of scientific investigations into WDs to support the discussion of his research. Another study in 2010 in the US on WDs by White [60] et al. also indicated a scarcity of publications on WD microbial contamination. Moreover, Hile [56] et al. corroborated the scarcity of publications on the microbial contamination of drinking water from WDs in the Eastern Coachella Valley, California in their studies. Zanetti [10] et al. indicated that little data on the bacteriological quality of water from WDs was available in Italy. Similarly, Liguori et al. in Italy [9] noted the scarcity of studies addressing water quality from WDs. The paucity of publications on WDs is a severe limitation to understanding the dynamics of microbial contamination and WDs, and to improving water quality from WDs. Bartram [53] et al., in their review on microbial contamination of non-household drinking water sources, determined that additional studies were needed to understand water quality status and provide guidelines for water monitoring and quality improvement. Baumgartner [6] et al. have counted the lack of studies on WDs as a hampering factor to water quality improvement in Switzerland. With substantially more studies on WDs, lawmakers may be able to make more informed decisions, generate policies, and allocate funding to communities affected by drinking water contaminants nationally and internationally. Many gastrointestinal infections related to WDs are unreported, and water-born outbreaks related to WD contamination with pathogenic microorganisms, such as *E. coli, P. aeruginosa*, and *Enterococcus faecalis*, remain undocumented and understudied, despite the exponential increase of drinking water consumption from WDs. Additional studies are needed to support better regulations and monitoring of drinking water. Although intestinal bacteria (e.g., coliforms) are rare in well-maintained municipal systems, several pathogens have been associated with WDs. These include *P. aeruginosa*, linked to respiratory and urinary infections; *Stenotrophomonas maltophilia*, associated with respiratory tract infections in immunocompromised individuals; *Enterococcus faecalis*, associated with gastrointestinal illness; and *Mycobacterium avium complex*, associated with pulmonary infections. The risk is particularly relevant in communities accustomed to consuming untreated water, where baseline exposure and susceptibility profiles may differ [31],[32],[48].

### 3.9. Recommendations

Conventional water dispensers generally rely on one or more of the following treatment processes: Activated carbon filtration, sediment filtration, microfiltration, reverse osmosis, UV disinfection, and ion exchange. However, the absence of a disinfectant residual means that post-treatment microbial regrowth remains possible, particularly when maintenance and filter replacement schedules are not properly followed. Based on the studies reviewed, in certain underserved regions where WVMs are commonly used as alternatives to municipal supply, the filtration systems of WDMs have not consistently ensured improved drinking water quality compared to tap water. Therefore, our recommendations focus on improving monitoring, maintenance practices, and the integration of antimicrobial materials and technologies specifically in these contexts. We can infer from the multiple studies reviewed in this paper that the filtration systems used in water vending machines (WVMs) often fail to consistently provide safe drinking water. Therefore, we propose the enforcement of stronger water quality monitoring practices, as well as the integration of new treatment technologies. To improve the quality of drinking water from WDs and mitigate microbial contamination, it is essential to implement materials and technologies that inhibit biofilm formation. The use of internal system lines made from biofilm-resistant materials or those coated with nanoparticles that discourage bacterial attachment and growth should help to improve the overall quality of water [70]. It is important to note that the implementation of nanoparticle-coated components must ensure material stability and safety. Researchers suggest that when nanoparticles are embedded or chemically bonded to internal surfaces, the risk of leaching is significantly reduced, even during prolonged use [70],[71]. However, systems that rely on loosely bound or free nanoparticles may pose potential release risks. Therefore, the adoption of such technologies should be accompanied by compliance with regulatory safety standards, long-term stability testing, and certification to confirm that no harmful nanoparticle migration occurs during operation. Additionally, periodic disinfection protocols, such as the application of hydrogen peroxide, have proven effective in significantly reducing microbial populations in microfiltered dispensers, as demonstrated by Sacchetti et al. [10]. These combined strategies address the prevention and control of microbial contamination within WDs. Regular maintenance, routine cleaning, and user hygiene practices are critical in maintaining water quality in WDs. Without consistent upkeep, even the most advanced systems may become prone to contamination. Quality control measures by regulatory authorities and adherence to maintenance schedules by owners are equally important. Materials used in WDs play a significant role in contamination prevention. For instance, nanoparticle-coated internal components not only resist biofilm formation but also reduce the likelihood of bacterial growth. Thus, material selection, when combined with proper disinfection protocols, provides an integrated approach to minimizing contamination. Our findings suggest that even in areas where tap water quality is compromised, WVMs may fail to consistently provide relatively safer drinking water. The studies reviewed indicate that although WVMs are intended to improve water quality through filtration, their performance depends heavily on maintenance, sanitation practices, filter replacement schedules, and biofilm control within the dispensing systems. When these are inadequate, WVMs can introduce or enable the regrowth of microorganisms, leading to contamination levels that may equal or exceed those found in the original tap water source.

## Use of AI tools declaration

The authors declare they have not used Artificial Intelligence (AI) tools in the creation of this article.
